# Diffuse Alveolar Hemorrhage Due to Malignant Arterial Hypertension – an Unusual Manifestation of a Common Disease

**DOI:** 10.5334/jbr-btr.959

**Published:** 2016-02-19

**Authors:** Daniel Ramos-Andrade, Francisco Silva, Andrea Canelas, Luís Curvo-Semedo, Filipe Caseiro-Alves

**Affiliations:** 1 CHUC, PT

**Keywords:** Malignant Hypertension, Diffuse alveolar hemorrhage

## Abstract

Diffuse alveolar hemorrhage is a clinicopathological syndrome that often leads to respiratory failure, with associated hemoptysis and anemia. Chest radiograph presents non-specific findings of perihilar infiltrates, while computed tomography shows ground-glass attenuation or areas of consolidation with interlobular septal thickening. Bronchoalveolar lavage is used to confirm the clinical and radiological suspicion.

While vasculitis and other causes of pulmonary renal syndrome are the most common causes of diffuse alveolar hemorrhage, malignant hypertension should be considered in the proper clinical setting. We present a case report of a 51-year-old previously healthy patient that was diagnosed with diffuse alveolar hemorrhage and acute renal failure due to malignant hypertension, through clinical and radiological findings.

## Case report

A 51-year-old Caucasian male presented to the emergency department with a four-day history of dry cough, orthopnea and asthenia. He was a smoker (30 packs/year) but had no other known previous pathologies, in particular arterial hypertension.

## Clinical findings

At presentation, he was hypoxic on room air (94% oxygen saturation) and had bibasal auscultatory crackles, but had no other clinical signs of heart failure. He had features of malignant hypertension (blood pressure 220/130 mmHg) and his serum creatinine level was 8.02 mg/dl (with a creatinine clearance level of 13 ml/min). He had no hematuria, however. Other laboratory findings were unremarkable, besides elevated blood urea nitrogen (104 mg/dl), mild anemia (serum hemoglobin concentration of 11.2 g/dl) and hypokalemia (2.5 mmol/L).

**Figure 1:**
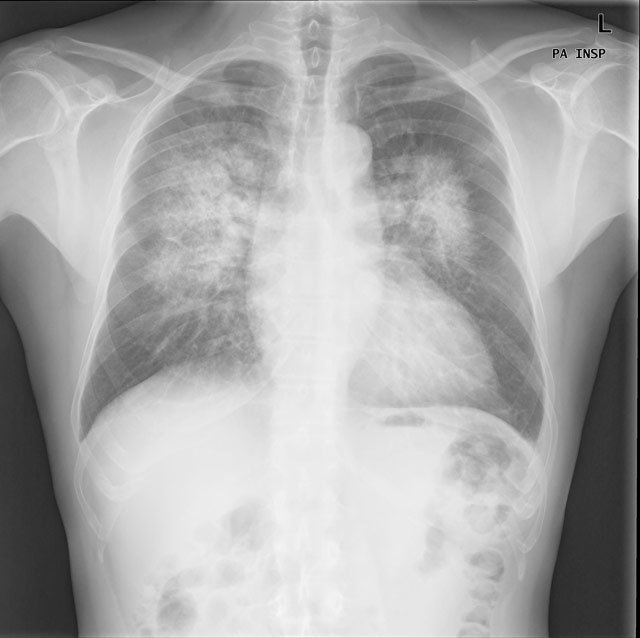
Chest radiograph (PA view) showing bilateral perihilar alveolar infiltrates. The heart size is not enlarged and there are no pleural effusions.

**Figure 2:**
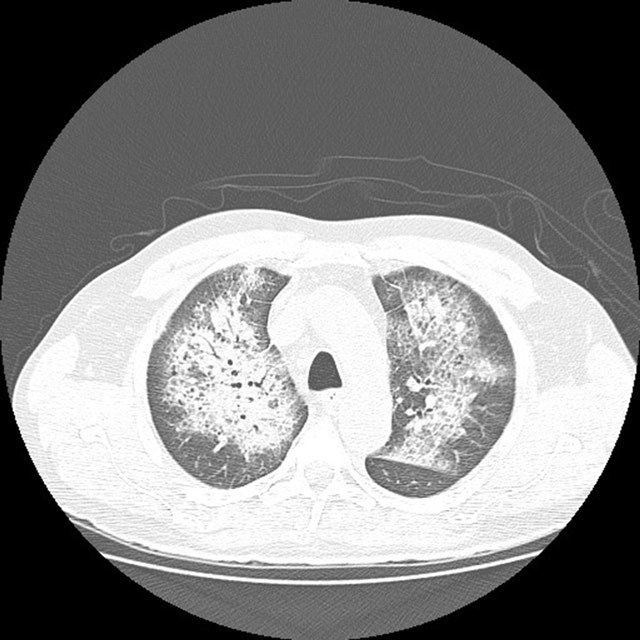
High resolution chest CT reveals interlobular septal thickening and ground-glass opacity in the upper perihilar areas, the so-called “crazy-paving” pattern.

Chest radiograph revealed bilateral perihilar areas of increased opacity. There were no radiologic signs of heart failure, like cardiomegaly or pleural effusions (Figure 1). High resolution chest computed tomography (CT) showed interlobular septal thickening and diffuse perihilar ground-glass attenuation with some areas of consolidation (Figure 2).

He underwent trachea-bronchial fibroscopy that revealed bronchial hyperemia and red bronchoalveolar lavage (BAL) fluid with increased numbers of red blood cells, which indicated diffuse alveolar hemorrhage.

The clinical presentation of lung hemorrhage and rapidly deteriorating renal function is usually due to a vasculitis or an immune-mediated pulmonary renal syndrome. Autoimmune blood tests were done, which excluded lupus, scleroderma, Churg-Strauss syndrome, Wegener granulomatosis and Goodpasture’s. A renal biopsy was also done, and revealed histopathological characteristics of hypertensive nephrosclerosis and also ruled out vasculitis and other causes of renal pulmonary syndrome.

Based on clinical, laboratory, radiological and BAL findings, malignant hypertension was diagnosed as the cause of the concomitant diffuse alveolar hemorrhage and renal failure. The patient was treated with oxygen administration, anti-hypertensive drugs and hemodialysis. Lung opacities started to clear within two days of presentation.

## Discussion

Diffuse alveolar hemorrhage is an increasingly recognized clinicopathological syndrome that may manifest as a life-threatening event. Histopathology reveals intra-alveolar red blood cells originating from the alveolar capillaries, fibrin and hemosiderin-laden macrophages [1,2].

Clinically, it is usually evidenced by the triad of hemoptysis, anemia and hypoxemic respiratory failure. Hemoptysis can be absent in one-third of the cases, like ours [1].

Chest radiography shows patchy or diffuse infiltrates, often with sparing of the apices, lung periphery and costophrenic angles [1,2].

High resolution lung CT revealed patchy or diffuse bilateral consolidations or ground-glass opacities in the acute phase. Air bronchograms are also common. Within a few days, interlobular septal thickening may become apparent. If ground glass opacities and interlobular septal thickening coexist, a crazy-paving pattern can be seen. Complete clearing of air-space and interstitial opacities can be expected within 10 days of the acute episode [1,2].

Making a specific diagnosis with radiography and CT findings alone is very difficult, as the crazy-paving pattern can also be found in other acute settings such as pulmonary edema (the cardiac silhouette is enlarged and there are associated pleural effusions), *Pneumocystis jirovecci* pneumonia (the patient is immunocompromised and has fever and increased inflammatory markers), ARDS (the lung infiltrates have peripheral distribution and slower clearing) or radiation pneumonitis (the lung disease is localized in the tissue within the radiation field) [3].

BAL usually confirms the clinical suspicion of alveolar hemorrhage when lavage aliquots are progressively more hemorrhagic. Hemosiderin-laden macrophages can also be found [1,2,4]. Once the diagnosis of diffuse alveolar hemorrhage is achieved through BAL, the underlying cause must be rapidly established so that adequate treatment can be chosen. There are several culprits, the most frequent being Wegener granulomatosis (32%), Goodpasture syndrome (13%), idiopathic pulmonary hemosiderosis (13%), collagen vascular diseases (13%) and microscopic polyangiitis (9%) [1]. Thoracoscopic or open lung biopsy may be considered in cases where the autoimmune panel is negative [1,2].

Malignant hypertension is characterized by elevated blood pressure accompanying encephalopathy or acute nephropathy as target organ damage. To the best of our knowledge, there have only been five reports of malignant-phase hypertension manifesting with diffuse alveolar hemorrhage [4,5,6,7,8]. The mechanism of how malignant hypertension causes lung hemorrhage is still unclear. While Hida et al. [5] hypothesized that the capillaries in the systemic circulation were injured by malignant hypertension, resulting in alveolar hemorrhage, Sato et al. [6] and Aithal et al. [7] postulated that left ventricular dysfunction caused by the arterial hypertension was the cause of pulmonary edema and the ensuing alveolar hemorrhage. In our case, however, the patient had no typical radiological findings of left ventricular failure.

In conclusion, although it is very rare for malignant hypertension to first manifest with pulmonary disease, when applicable, this diagnosis should be included in the differential of acute diffuse alveolar hemorrhage.

## Competing Interests

The authors declare that they have no competing interests.
